# Balance Training With a Vibrotactile Biofeedback System Affects the Dynamical Structure of the Center of Pressure Trajectories in Chronic Stroke Patients

**DOI:** 10.3389/fnhum.2019.00084

**Published:** 2019-03-12

**Authors:** Kentaro Kodama, Kazuhiro Yasuda, Nikita A. Kuznetsov, Yuki Hayashi, Hiroyasu Iwata

**Affiliations:** ^1^Department of Economics, Kanagawa University, Yokohama, Japan; ^2^Research Institute for Science and Engineering, Waseda University, Tokyo, Japan; ^3^School of Kinesiology, Louisiana State University, Baton Rouge, LA, United States; ^4^Graduate School of Creative Science and Engineering, Waseda University, Tokyo, Japan

**Keywords:** stroke, postural control, haptic biofeedback, balance rehabilitation, detrended fluctuation analysis (DFA)

## Abstract

Haptic-based vibrotactile biofeedback (BF) is a promising technique to improve rehabilitation of balance in stroke patients. However, the extent to which BF training changes temporal structure of the center of pressure (CoP) trajectories remains unknown. This study aimed to investigate the effect of vibrotactile BF training on the temporal structure of CoP during quiet stance in chronic stroke patients using detrended fluctuation analysis (DFA). Nine chronic stroke patients (age; 81.56 ± 44 months post-stroke) received a balance training regimen using a vibrotactile BF system twice a week over 4 weeks. A Wii Balance board was used to record five 30 s trials of quiet stance pre- and post-training at 50 Hz. DFA revealed presence of two linear scaling regions in CoP indicating presence of fast- and slow-scale fluctuations. Averaged across all trials, fast-scale fluctuations showed persistent dynamics (α = 1.05 ± 0.08 for ML and α = 0.99 ± 0.17 for AP) and slow-scale fluctuations were anti-persistent (α = 0.35 ± 0.05 for ML and α = 0.32 ± 0.05 for AP). The slow-scale dynamics of ML CoP in stroke patients decreased from pre-training to post-BF training (α = 0.40 ± 0.13 vs. 0.31 ± 0.09). These results suggest that the vibrotactile BF training affects postural control strategy used by chronic stroke patients in the ML direction. Results of the DFA are further discussed in the context of balance training using vibrotactile BF and interpreted from the perspective of intermittent control of upright stance.

## Introduction

Following a stroke, a complex interplay of sensory, motor, and cognitive impairments may interfere with balance (de Haart et al., 2004). Stroke patients commonly show increased postural sway and asymmetric weight distribution while standing (Mansfield et al., 2013; Hendrickson et al., 2014). Impaired balance decreases mobility and increases fall risk in elderly stroke patients (Lamb et al., 2003). Vibrotactile biofeedback (BF) application to the trunk is a promising method for restoring balance ability (e.g., Dozza et al., 2007; Bechly et al., 2013). However, we previously found that a 4 week vibrotactile BF training did not induce significant changes on several center of pressure (CoP) measures (i.e., sway area, path length) in chronic stroke patients (Yasuda et al., 2018).

In this report, we apply *detrended fluctuation analysis* (DFA; Peng et al., 1994) to characterize the effects of this BF training in stroke patients. DFA offers an additional perspective on postural control dynamics in comparison to traditional CoP metrics because it examines control processes across multiple time scales (Eke et al., 2002; Seuront, 2009). DFA can evaluate presence of temporal correlations across a range of window sizes (Brown and Liebovitch, 2010). Fractal processes can be categorized in two families: *fractional Gaussian noise* (fGn) and *fractional Brownian motions* (fBm). The scaling exponent, DFA α, is interpreted as an indicator of temporal correlation pattern: If 0 < α < 1 (fGn) with anti-persistent (α < 0.5), random (α = 0.5), or persistent dynamics (α > 0.5). If 1 < α < 2 (fBm) with under-diffusive (a < 1.5), Brownian (α = 1.5), hyper-diffusive dynamics (α > 1.5) (Delignières et al., 2011).

Previous studies have indicated that DFA can identify differences in postural control strategy between young and elderly adults (Amoud et al., 2007; Duarte and Sternad, 2008). Roerdink et al. (2006) applied DFA to CoP data to compare stroke patients with healthy elderly and showed that the CoP trajectories of both the healthy elderly and stroke patients exhibited temporally correlated patterns rather than random noise (Roerdink et al., 2006).

The dynamical structure of CoP during quiet stance is characterized by presence of multiple scaling regions (Minamisawa et al., 2009; Teresa Blázquez et al., 2009; Kuznetsov et al., 2013). Kuznetsov et al. (2013) reported three scaling regions in a sample of healthy young adults. Presence of multiple scaling regions may be indicative of intermittent control strategy (Loram et al., 2011) or continuous open- and closed-loop control strategy (Collins and De Luca, 1995).

The effect of vibrotactile BF on the dynamics across multiple-scales for postural control remains unknown however. Postural control strategy used by stroke patients may differ from the strategies used by younger adults or healthy elderly due to freezing, asymmetrical weight distribution, and sensory input alterations. We hypothesized that intensive balance training using vibrotactile BF would affect the dynamical structure of CoP trajectories in chronic stroke patients.

## Materials and Methods

### Participants

We recruited 9 participants with chronic hemiparetic stroke from the Department of Physical Medicine and Rehabilitation, Tokyo General Hospital (Table 1). Inclusion criteria were positive history of chronic unilateral ischemic or hemorrhagic stroke, age 50–80 years, stroke >6 months ago, completion of conventional therapy, and ability to stand unsupported for 10 min and sense BF system vibrations. Prior to the study, all participants underwent conventional balance rehabilitation with a physical therapist twice a week.

**Table 1:** Participants' demographic data (*n* = 9).

**Participant**	**Gender**	**Age**	**Type of stroke**	**Time since stroke**	**Hemiplegic side**	**Brs (L/E)**	**Superficial sensation (L/E)**
1	M	75–80	Infarcted	2 years 1 month	L	V	Mild
2	F	75–80	Infarcted	10 years 7 months	R	IV	Mild
3	M	55–60	Hemorrhagic	4 years	R	V	Moderate
4	M	55–60	Hemorrhagic	4 years 8 months	L	IV	Mild
5	M	50–55	Hemorrhagic	8 years	R	IV	Mild
6	M	75–80	Infarcted	7 years	L	IV	Moderate
7	M	55–60	Hemorrhagic	8 years 5 months	R	IV	Moderate
8	F	65–70	Hemorrhagic	13 years 5 months	R	III	Severe
9	M	65–70	Infarcted	3 years	L	V	Mild

*Brs, Brunnstrom recovery stage; L/E, lower extremity; M, male; F, female*.

### BF System Overview

**Figure 1:**
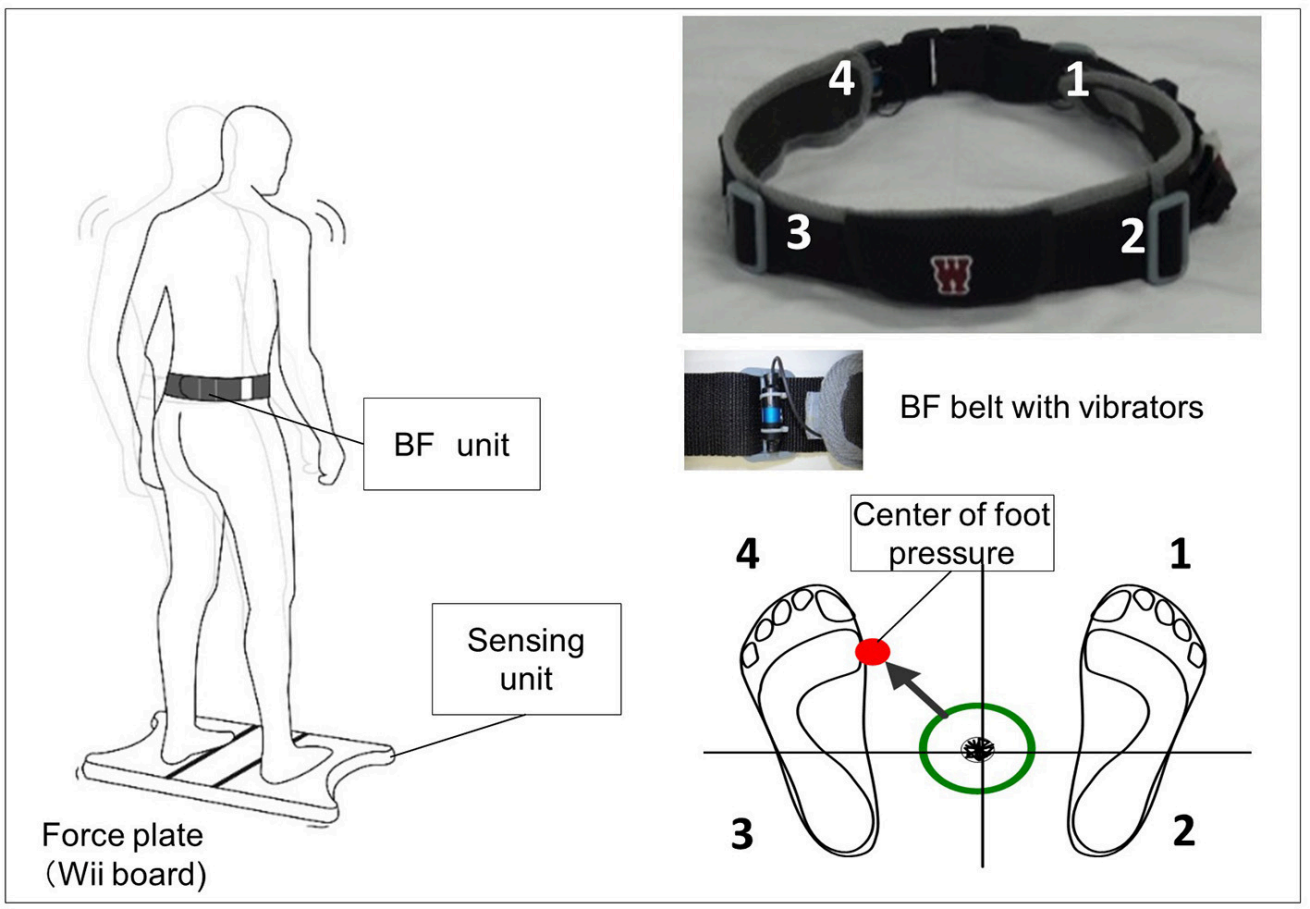
Biofeedback system overview. Vibrators on the pelvic belts worn by the participants vibrated in the corresponding direction when the center of pressure (CoP) exceeded the predefined threshold (e.g., if CoP shifts to the back left, the back left vibrator is activated).

The vibrotactile BF device consisted of a Nintendo Wii balance board (Nintendo Co., Ltd., Kyoto, Japan) and a personal computer with custom software (Visual Studio; Microsoft Corp., Redmond, WA, USA). CoP position data were measured in both the ML and AP directions at 50 Hz. The system uses vibration motors attached to the belt at the level of the pelvic girdle (bilaterally attached at the anterior superior iliac and posterior superior iliac spine) to convey information about body sway (Figure 1).

### Protocol and Postural Task

Participants underwent 45 min of BF training 2 times per week for 2 weeks. The training consisted of two task-oriented balance training exercises used as part of the conventional rehabilitation (Teasell et al., 2008).

Two balance training exercises were used: (1) standing on a rubber foam mat (balance mat, Sanwa Kako Co. Ltd, Japan): participants stood barefoot on the mat with their eyes open and were instructed to use the BF information to stabilize their postural sway (i.e., they were instructed to stay within the predefined threshold area using BF information) and (2) weight-shifting to the paralyzed limb: participants were instructed to move their paralyzed lower limb forward and then put their weight on that limb. While doing so, participants used the BF information to help maintain a stable standing position. Each training session comprised 10 repetitions of the balance task (1 min per repetition, 10 min total) with a short interval between repetitions. The BF threshold setting was reset on each day of training before implementing tasks (1) and (2). We determined the circular threshold as a 95% confidence circle area (Yasuda et al., 2017) during the 30 s stance. Target area was defined as 90% of the pre-measured 95% confidence circle area. The BF vibrators were activated when the CoP exceeded this threshold (Yasuda et al., 2017).

### Analysis

**Figure 2:**
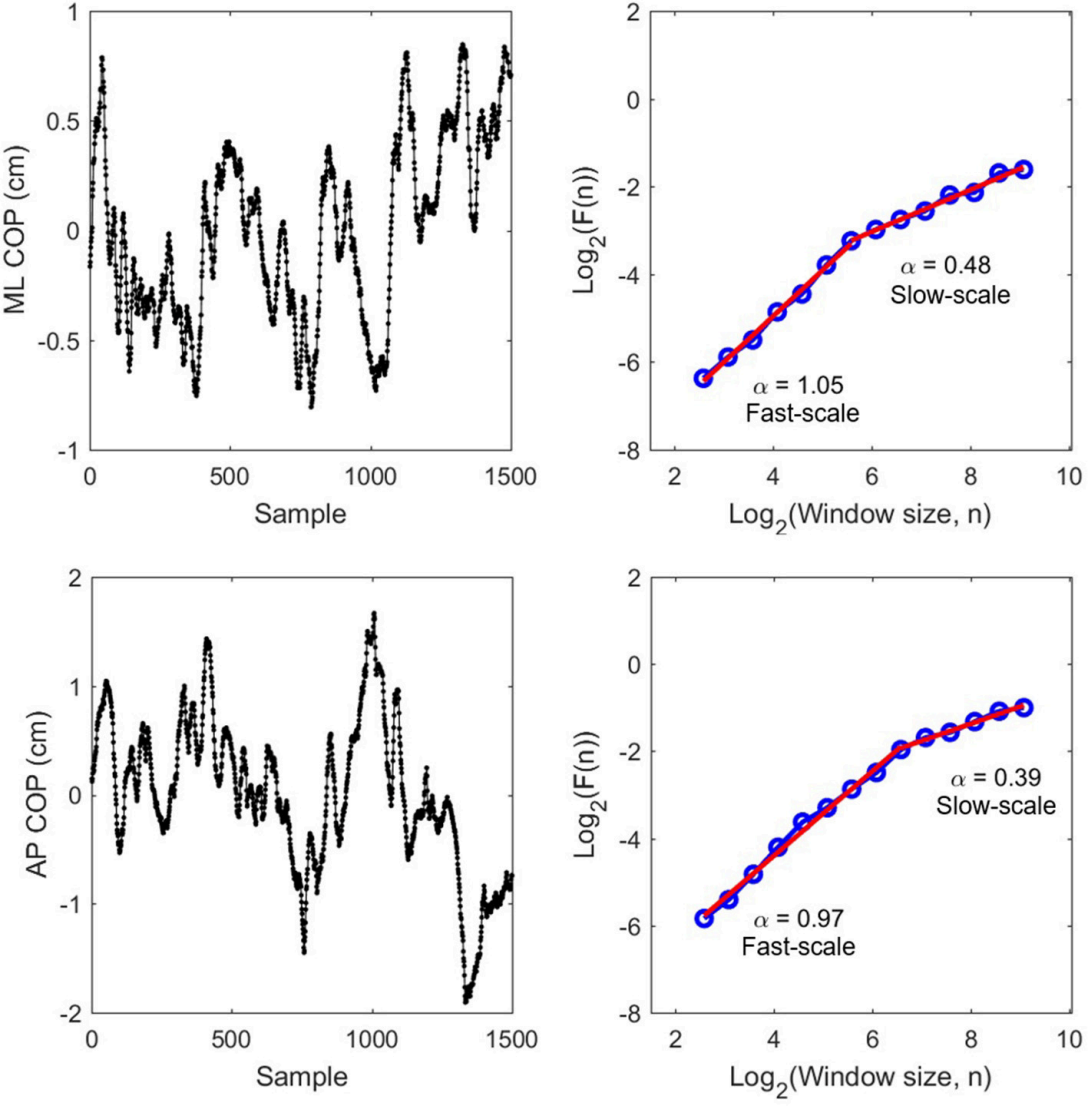
Raw COP data **(Left)** for ML and AP and their corresponding DFA plots **(Right)** from P8 pre-test trial 2. Red slopes indicate estimated scaling exponents α for fast-scale and slow-scale fluctuations.

Traditionally, DFA requires integration of the signal if it is similar to an fGn process. COP variability is non-stationary and is therefore not like an fGn—a stationary process. Hence, COP fluctuations are already more similar to fBm and do not require integration prior to DFA. The range of scales considered ranged from 0.12 s to 10.86 s. The evaluated scales were generated as *Scale* = 2^*w*^/F_s_, where F_s_ = 50 Hz and *w* ranged from log_2_(6 samples) to log_2_(750 samples) in increments of 0.5 on log_2_ scale (e.g., *w* = 2.585, 3.085, 3.585, …, 9.085) for a total of 14 scales in the range (F_s_: sampling frequency). Using these scales allowed us to have equal logarithmic distance between the windows on the DFA plot (see Figure 2 for an illustration).

CoP was filtered using the Savitzky-Golay filter (order 3, length 7) to minimize distortions associated with linear filtering techniques (Gao et al., 2011). A linearity test was performed comparing fit of multiple models to the DFA plots (Ton and Daffertshofer, 2015). The results showed that a linear model with a single scaling exponent was not a good fit for our data. However, automated fits suggested a range of models: quadratic, cubic, exponential, and 2- and 3-region linear models in different individual trials.

Based on preliminary visual inspection of all DFA plots of all COP recordings, we made the assumption that the multi-scale dynamics could be adequately characterized using a two-region linear model. We chose to fit a linear (vs. polynomial or exponential) model because it allows to interpret DFA slopes based on the fBm/fGn model (persistent, anti-persistent, random). We chose a 2-region (vs. 3-region) model because previous work has identified 2 scaling regions in COP (Collins and De Luca, 1995 and Kuznetsov et al., 2013; see their results when downsampled to 50 Hz).

We identified the cross-over point between the two regions based on visual inspection of each DFA plot. For the AP signals Region 1 ranged from 0.12 s to 1.37 s (fast-scale fluctuations) and Region 2 ranged from 1.37 to 10.86 s (slow-scale fluctuations). For the ML signals region 1 ranged from 0.12 s to 1.79 s and Region 2 is ranged from 1.79 to 10.86 s.

Scaling exponents were calculated for each region and a paired *t*-test was used to compare pre- and post-BF training in the ML and in the AP directions.

## Results

**Figure 3:**
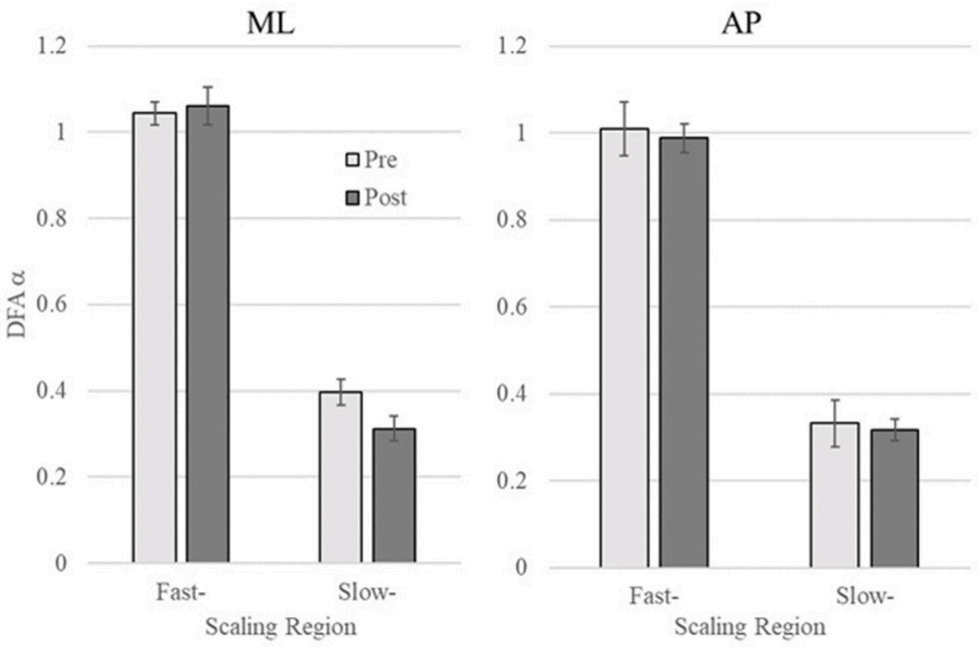
DFA scaling exponent of pre- and post-training in the mediolateral (ML) and anteroposterior (AP) directions **(Left** and **Right**, respectively). Error bars indicate standard error of the mean.

To examine whether vibrotactile BF training affect the CoP dynamics in chronic stroke patients, the DFA was applied to CoP trajectories data in the ML and AP directions. Figure 2 shows a representative DFA plots in a single trial. Table 2 presents the average DFA scaling exponents for each participant. Figure 3 shows the mean and standard error of the DFA scaling exponents for fast and slow-scales in the ML and AP directions.

**Table 2:** DFA scaling exponent α for two scaling regions.

	**ML**				**AP**			
	**Region 1**		**Region 2**		**Region 1**		**Region 2**	
**Participant**	**Pre**	**Post**	**Pre**	**Post**	**Pre**	**Post**	**Pre**	**Post**
1	1.10	1.10	0.57	0.40	1.06	0.99	0.40	0.24
2	1.22	1.19	0.25	0.21	0.96	1.07	0.17	0.29
3	1.01	1.09	0.36	0.35	1.23	1.01	0.38	0.29
4	1.04	0.94	0.58	0.36	0.79	0.77	0.40	0.36
5	1.07	1.11	0.48	0.30	1.11	1.04	0.50	0.31
6	0.96	1.05	0.35	0.33	0.86	1.00	0.31	0.28
7	0.99	1.02	0.23	0.18	1.35	1.27	0.34	0.45
8	1.01	0.91	0.31	0.25	0.96	0.84	0.31	0.29
9	1.00	1.14	0.43	0.43	0.76	0.90	0.18	0.36
Mean	1.04	1.06	0.40	0.31	1.01	0.99	0.33	0.32
SD	0.08	0.09	0.13	0.09	0.20	0.14	0.11	0.06

ML COP: There was no significant difference between the DFA scaling exponents in the pre- and post-training in Region 1 (*p* = 0.56), while the scaling exponent was lower post-training (α = 0.31 ± 0.09) compared to pre-training (α = 0.40 ± 0.13) in Region 2, *t*_(8)_ = 3.06, *p* = 0.015 (see Figure 3).

AP COP: There were no significant differences between pre- and post-training in both Region 1 and 2 (*p* = 0.62 and *p* = 0.75, respectively).

## Discussion

To the best of our knowledge, this is the first study to describe changes in the dynamical structure of CoP trajectories resulting from vibrotactile feedback training. Our results showed that CoP variability during quiet stance is characterized by two linear scaling regions in chronic stroke patients. The first scaling region captures relatively fast-scale CoP dynamics that range from 0.12 to 1.37 s for AP CoP and from 0.12 to 1.79 s for ML CoP. The second region captures relatively slow-scale dynamics ranging from 1.37 to 10.86 s for AP and from 1.79 to 10.86 s for ML. DFA scaling exponents indicate that the fast-scale region is characterized by persistent CoP fluctuations, while the slow-scale region is characterized by anti-persistent CoP fluctuations.

Such dynamics can be interpreted from the perspective of intermittent control (Loram et al., 2011), where the position of the center of gravity is allowed to drift with only intermittent corrections as long as it remains within the boundaries of the base of support (BOS). This kind of control strategy would account for the observation of persistent fluctuations at the fast time scales (drifting toward the boundary) and anti-persistent fluctuations at slow scales (intermittent correction away from the boundary). This is also consistent with the rambling-trembling hypothesis of postural control (Zatsiorsky and Duarte, 2000). According to this hypothesis, CNS controls upright posture using two hierarchical levels: a reference position for maintaining equilibrium is specified at one level, and the lower level maintains balance around that reference position by negating deviations (“trembling”) from it. The set point for equilibrium migrates (“rambles”) during quiet stance. The fast-scale persistent region in our results may be capturing the trembling component while the anti-persistent slow-scale region may be indexing changes in the rambling dynamics.

Taken from this perspective, our results indicate that the BF training induced a change in the error correction strategy in ML CoP because the slow-scale scaling exponent suggested stronger anti-persistent dynamics in post-training compared to pre-training. We interpret reduction as tighter control over body sway as it gets closer to the BOS limits. For stroke patients it is often difficult to maintain postural balance, particularly in the ML direction because of hemiplegia. This can cause asymmetric balancing referred to as the weight-bearing asymmetry (WBA), destabilizing coordination between the left and right limbs, and large variations in CoP. During quiet stance, a substantial amount of WBA in favor of the non-paretic leg is commonly observed. Therefore, we speculate that intensive balance exercise might show stronger effects particularly in the ML direction rather than that in the AP direction.

Roerdink et al. (2006) reported that the scaling exponent was not affected by standard rehabilitation. In contrast, our results suggest that the BF training can affect the scaling exponent in slow-scale region of the CoP trajectories of stroke patients in the ML direction. These results may suggest that the BF training has a potential to lead the change in the CoP dynamics beyond that of typical rehabilitation.

We previously found that 4 week vibrotactile BF training did not induce significant changes in traditional CoP measures (i.e., sway area, mean velocity) in chronic stroke patients (Yasuda et al., 2018). Thus, it is possible that vibrotactile BF may affect only the temporal structure of CoP trajectories. This possibility is worth considering in clinical settings because it is important to evaluate the effect of BF devices on the CoP dynamics. Although the underlying mechanism remains unclear, we speculated that intensive subtle coordination of the CoP within the BF circular threshold may influence the CoP dynamics. Further experimental studies (e.g., comparison of the different sizes of the threshold area) are warranted to specifically describe the expected effect of the BF system.

Although these results should be interpreted cautiously, the present report has important implications because the results describe the specific influence of BF devices by applying dynamical methods (e.g., DFA). Importantly, persistent dynamics in the fast scaling region do not signify presence of long-range correlations in these data—a much longer durations of trials are required to establish long-range correlations (Duarte and Zatsiorsky, 2001). One limitation of the study is the lack of a control group. However, the internal validity was strengthened by excluding participants who had experienced a stroke <6 months before the study. Therefore, the results may have been biased by the learning effects. Further studies should be assessed with more rigorous methodology or randomized study designs.

## Ethics Statement

All participants provided informed consent. All procedures were approved by the Ethics Committee for Human Research, Waseda University.

## Author Contributions

KY designed this study, acquired and analyzed the data, and drafted the manuscript. KK and NK substantially contributed to data analysis and manuscript drafting. YH contributed to data acquisition and analysis. HI helped conceive the BF system and design the study. All authors have read and approved the final manuscript. No one who qualifies for authorship has been omitted from the list.

### Conflict of Interest Statement

The authors declare that the research was conducted in the absence of any commercial or financial relationships that could be construed as a potential conflict of interest.

## Appendix

Brunnstrom stages of stroke recovery.

Lower extremity function:

Stage I: Flaccidity.
Stage II: Minimal voluntary movements.
Stage III: Hip flexion, knee flexion, and ankle dorsiflexion performed as a combined motion while sitting and standing.
Stage IV: While sitting: knee flexion beyond 90°; ankle dorsiflexion with the heel on the floor.
Stage V: While standing: isolated knee flexion with hip extended; isolated ankle dorsiflexion with knee extended.
